# The Effects of Brazilian Green Propolis against Excessive Light-Induced Cell Damage in Retina and Fibroblast Cells

**DOI:** 10.1155/2013/238279

**Published:** 2013-12-12

**Authors:** Hiromi Murase, Masamitsu Shimazawa, Mamoru Kakino, Kenji Ichihara, Kazuhiro Tsuruma, Hideaki Hara

**Affiliations:** ^1^Molecular Pharmacology, Department of Biofunctional Evaluation, Gifu Pharmaceutical University, 1-25-4 Daigaku-nishi, Gifu 501-1196, Japan; ^2^Nagaragawa Research Center, Api Co., Ltd., 692-3 Nagara, Gifu 502-0071, Japan

## Abstract

*Background*. We investigated the effects of Brazilian green propolis and its constituents against white light- or UVA-induced cell damage in mouse retinal cone-cell line 661W or human skin-derived fibroblast cells (NB1-RGB). *Methods*. Cell damage was induced by 3,000lx white light for 24 h or 4/10 J/cm^2^ UVA exposure. Cell viability was assessed by Hoechst33342 and propidium iodide staining or by tetrazolium salt (WST-8) cell viability assay. The radical scavenging activity of propolis induced by UVA irradiation in NB1-RGB cells was measured using a reactive-oxygen-species- (ROS-) sensitive probe CM-H_2_DCFDA. Moreover, the effects of propolis on the UVA-induced activation of p38 and extracellular signal-regulated kinase (ERK) were examined by immunoblotting. *Results*. Treatment with propolis and two dicaffeoylquinic acids significantly inhibited the decrease in cell viability induced by white light in 661W. Propolis and its constituents inhibited the decrease in cell viability induced by UVA in NB1-RGB. Moreover, propolis suppressed the intracellular ROS production by UVA irradiation. Propolis also inhibited the levels of phosphorylated-p38 and ERK by UVA irradiation. *Conclusion*. Brazilian green propolis may become a major therapeutic candidate for the treatment of AMD and skin damage induced by UV irradiation.

## 1. Introduction

People are exposed to visible light or ultraviolet (UV) on a daily basis. When exposed excessively, they will experience serious effects in their eyes or skin. Skin is the only organ that is directly exposed to UV irradiation. The skin coexists with many environmental pollutants that are oxidants themselves or can catalyze the formation of reactive oxygen species (ROS). Oxidative damage to the skin, induced by several exogenous and endogenous factors, such as ultraviolet (UV) irradiation, tobacco smoke, infrared radiation, transition metal ions, and enzymatic and nonenzymatic antioxidant impairment, has been acknowledged as a key factor of intrinsic and photoinduced skin aging. Ultimately, it induces actinic elastosis and skin cancer [1].

We can classify the skin aging process into intrinsic aging and photoaging. Damage to human skin resulting from repeated exposure to UV irradiation (photoaging) and damage caused by the passage of time, cell replication, and aerobic metabolism (intrinsic aging) are considered to be distinct entities rather than similar skin aging processes [2]. Sunlight consists of the infrared, visible, and ultraviolet regions of the spectrum. Ultraviolet radiation can be classified under UVA, UVB, and UVC wavelengths [3]. UVC (200–280 nm) is blocked by the ozone layer. UVA (320–400 nm) and UVB (280–320 nm) can pass through the ozone layer, cross the epidermis, and reach the dermis. UVA waves have many biological efficacies on living organisms. UVA induces the production of matrix metalloproteinases that deteriorate the extracellular matrix. UVA also induces the production of singlet oxygen, which causes eliminations or point mutations in mitochondrial DNA and is involved in DNA damage, which activates the DNA damage response system, finally leading to cell senescence. Therefore, UVA is considered a fundamental cause of aging.

High levels of visible light or UV may cause ocular damage, especially later in life. It has been noted that long-term light exposure results in photoreceptor degradation, and it may be among the most relevant damaging factors involved in age-related macular degeneration (AMD) [4]. Excessive light exposure can be a risk factor for the onset and progression of AMD [5] and it leads to photoreceptor degeneration in animals [6, 7]. Both external and internal factors are thought to be a pathogenesis of AMD [8, 9], and exposure to sunlight or ultraviolet radiation is also a well-established risk factor for AMD.

Propolis is made from a sticky substance that honeybees produce by mixing their own waxes with resinous sap obtained from the bark and leaf-buds of certain trees and other flowering plants. Propolis is used as a sealant and sterilant in honeybee nests. The color of propolis can be green, yellow, brown, or almost black depending on the plants from which the resinous substance is collected [10]. The properties and constituents of propolis also differ with its geographical origin [11]. Brazilian green propolis is made of aromatic acids (cinnamic acid derivatives, ferulic acid, and caffeic acid), diethyl methyl succinate, isobutylquinoline, general acetal, patchouli alcohol, menthol, amyrins, and flavonoids. Brazilian propolis has been the subject of many studies due to its biological activities, such as its antibacterial [12, 13], antifungal [11, 14–17], antiviral [18, 19], anti-inflammatory [20], antioxidative [21], hepatoprotective [22], tumoricidal [23], and antiangiogenesis activities [24], as well as its neuroprotective activities against oxygen-glucose deprivation stress [25]. Furthermore, propolis and its compounds, caffeic acid phenethyl ester (CAPE), and chrysin may restrain cell cycle proliferation or induce apoptosis in tumor cells [26].

The purpose of the present study was to clarify the effects of Brazilian green propolis and its constituents against visible light- or UVA-induced cell damage in 661W photoreceptor cells or human skin-derived fibroblasts.

## 2. Materials and Methods

### 2.1. Materials

The drugs and sources used were as follows: Dulbecco's modified Eagles' medium (DMEM), phenol red-free DMEM with sodium pyruvate without L-Glutamine, and dimethyl sulfoxide (DMSO) and were purchased from Nacalai Tesque Inc. (Kyoto, Japan). Penicillin and streptomycin were purchased from Meiji Seika Kaisha Ltd. (Tokyo, Japan). Fetal bovine serum (FBS) was purchased from VALEANT (Costa Mesa, CA, USA). The cell counting Kit-8 (WST-8) was purchased from Dojin Kagaku (Kumamoto, Japan). Hoechst 33342, propidium iodide (PI), and 5-(and-6-)chloromethyl-2′,7′-dichlorodihydrofluorescein diacetate acetyl ester (CM-H_2_DCFDA) were purchased from Molecular Probes (Eugene, OR, USA). 3,4-Di-*O*-caffeoylquinic acid (3,4-CQA), 3,5-di-*O*-caffeoylquinic acid (3,5-CQA), *p*-coumaric acid, and chlorogenic acid were kindly gifted by Api Co., Ltd. (Gifu, Japan). The propolis used in the present study was Brazilian green propolis (Minas Gerais State, Brazil), which originates mainly from *Baccharis dracunculifolia*. The *Baccharis* propolis was extracted with water at 50°C to yield the extract used here (water extract of Brazilian green propolis; WEP). The main constituents of WEP were previously reported.

### 2.2. Cell Cultures

The mouse retinal cone-cell line 661W, a transformed mouse cone-cell line derived from mouse retinal tumors, was a gift from Dr. Muayyad R. Al-Ubaidi (University of Oklahoma Health Sciences Center, Oklahoma City, OK, USA). The 661W cells were maintained in DMEM containing 10% fetal bovine serum (FBS), 100 U/mL penicillin, and 100 *μ*g/mL streptomycin. Normal human skin fibroblast cells (NB1-RGB) were purchased from the RIKEN Bioresource Center Cell Bank (Tsukuba, Ibaraki, Japan). Cells were cultured in phenol red-free DMEM with sodium pyruvate, without L-Glutamine, containing 10% FBS, 100 U/mL penicillin, and 100 *μ*g/mL streptomycin. Both cultures were maintained at 37°C in a humidified atmosphere of 95% air and 5% CO_2_. The 661W and NB1-RGB cells were passaged by trypsinization every 3 to 4 days, respectively.

### 2.3. Exposure of 661W to White Light

The origin of the 661W cell line is a mouse retinal tumor. 661W has been characterized as a cone-specific cell line that expresses cone blue opsin or green opsin, transducin, and arrestin [27]. The 661W cultures are useful for the estimation of light-induced stress in cone photoreceptors, because they are able to respond to light [28]. The 661W mouse retinal cone-cell line cells were seeded at a density of 1 × 10^3^ cells per well into a 96-well plate, and the cells were then incubated in a humidified atmosphere of 95% air and 5% CO_2_ at 37°C for 24 h. The entire medium was then replaced with phenol red-free DMEM containing 1% FBS. After replacement of the medium, propolis and its constituents were added to the culture. One h after the addition of reagents, the cultures were exposed to 3,000lx of white fluorescent light (C-FPS115D; Nikon, Tokyo, Japan) for 24 h at 37°C. The luminance was measured using an LM-332 light meter (As One, Osaka, Japan).

### 2.4. Exposure of 661W or NB1-RGB to UVA Irradiation

The 661W and NB1-RGB cultures were seeded at a density of 3 × 10^3^ and 1 × 10^3^ cells per well into 96-well plates, respectively, and the cells were then incubated in a humidified atmosphere of 95% air and 5% CO_2_ at 37°C for 24 h. To induce UVA stress, the 661W and NB1-RGB cells were washed with phenol red-free DMEM containing 1% FBS. After replacement of the medium, propolis and its constituents were added to the culture. One h after the addition of reagents, the 661W cultures were exposed to 4 J/cm^2^ of UVA light (365 nm UVA light source, CL-1000L UV Crosslinkers; Ultraviolet Products Ltd., Cambridge, UK), while the NB1-RGB cultures were exposed to 10 J/cm^2^. The UVA light was above the 96-well plate at a fixed distance of 11.5 cm. Control cells were incubated under the same conditions as experimental cells, but were not exposed to UVA because they were covered with aluminum foil.

### 2.5. Cell Proliferation Assay

To evaluate cell survival, we examined the change in fluorescence intensity that followed the cellular reduction of WST-8 to formazan. All experiments were performed in phenol red-free DMEM at 37°C. Cell viability was assessed by culturing cells in a culture medium containing 10% WST-8 (cell counting Kit-8) for 0 to 6 h at 37°C and was obtained by scanning with a microplate reader at 492 nm. This absorbance was expressed as a percentage of that in the control cells (which were in phenol red-free DMEM containing 1% FBS), after subtraction of background absorbance.

### 2.6. Cell Death Assay (Hoechst 33342 and PI Staining)

Cell death was observed by using combination staining with two fluorescent dyes, Hoechst 33342 and PI. To examine the effects of propolis on cell death induced by UVA irradiation, NB1-RGB cells were seeded at a density of 1,000 cells per well into 96-well plates. After pretreatment with propolis, the cells were irradiated with UVA 10 J/cm^2^. At the end of this culture period, Hoechst 33342 (excitation/emission wavelengths, 360/490 nm) or PI (excitation/emission wavelengths, 535/617 nm) was added to the culture medium for 15 min at final concentrations of 8 and 1.5 *μ*M, respectively. Images were collected using an epifluorescence microscope (IX70; Olympus, Tokyo, Japan) fitted with a charge-coupled device camera (DP30BW; Olympus) and fluorescence filters for Hoechst 33342 (U-MWU; Olympus) and PI (U-MWIG; Olympus).

### 2.7. Antioxidant Capacity Assay

NB1-RGB cells and 661W cells were seeded at a density of 1 × 10^3^ cells and 2 × 10^3^ cells per well into 96-well plates and then incubated in a humidified atmosphere of 95% air and 5% CO_2_ at 37°C, respectively. 24 h later, the cell culture medium was replaced before treatment with propolis or its vehicle (phenol red-free DMEM containing 1% FBS). After pretreatment with propolis or its vehicle for 1 h, we added the radical probe, 5-(and-6-)chloromethyl-2′,7′-dichlorodihydrofluorescein diacetate, and acetyl ester (CM-H_2_DCFDA) (10 *μ*M) by incubation for 20 min at 37°C. Then, the cell-culture medium was replaced to remove the extra probe. CM-H_2_DCFDA (inactive for ROS) is converted to dichlorofluorescein (DCFH) (active for ROS) by being taken into the cell and acted upon by an intracellular enzyme (esterase). To generate the ROS, we irradiated UVA 10 J/cm^2^ and 3,000lx of white fluorescent light for 24 h, respectively. Fluorescence was measured after the ROS-generating compounds had been present for 6 h after the UVA or white light irradiation using Skan It RE for Varioskan Flash 2.4 (Thermo Fisher Scientific, Waltham, MA, USA) at excitation/emission wavelengths of 485/535 nm.

### 2.8. Western Blot Analysis

NB1-RGB cells and 661W cells were washed with PBS, harvested, and lysed using a cell-lysis buffer (RIPA buffer R0278; Sigma-Aldrich) with protease (P8340; Sigma-Aldrich) and phosphatase inhibitor cocktails (P2850 and P5726; Sigma-Aldrich). The lysates were centrifuged at 12,000 ×g or 15 min at 4°C. The supernatants were collected and boiled for 5 min in SDS sample buffer (Wako). The protein concentration was measured by comparison with a known concentration of bovine serum albumin using a bicinchoninic acid (BCA) protein assay kit (Pierce Biotechnology, Rockford, IL, USA). A mixture of equal parts of an aliquot of protein and sample buffer with 10% 2-mercaptoethanol was subjected to 10% sodium dodecyl sulfate-polyacrylamide gel electrophoresis. The separated protein was then transferred onto a polyvinylidene difluoride membrane (Immobilon-P; Millipore Corporation, Bedford, MA, USA). The membranes were incubated with the following primary antibodies: phosphorylated p38 mouse monoclonal antibody (Promega, Madison, WI, USA) (1 : 1000), phosphorylated ERK rabbit polyclonal antibody (Cell Signaling Technology Inc., Danvers, MA, USA) (1 : 1000), p38 mouse monoclonal antibody (Santa Cruz Biotechnology Inc., Santa Cruz, CA, USA) (1 : 1000), ERK rabbit polyclonal antibody (Cell Signaling) (1 : 1000), and *β*-actin mouse monoclonal antibody (Sigma-Aldrich) (1 : 4000). After this incubation, the membrane was incubated with the secondary antibody: HRP-conjugated goat anti-rabbit IgG (Pierce Biotechnology) (1 : 2000). The immunoreactive bands were visualized using Super Signal West Femto Maximum Sensitivity Substrate (Pierce Biotechnology) and measured using GelPro (Media Cybernetics, Silver Spring, MD, USA). To measure the phosphorylation levels of ERK and p38, we normalized them with total ERK (t-ERK) and total p38 (t-p38), respectively.

### 2.9. Effects of a MAPK Inhibitor on UVA-Induced Cellular Damage

NB1-RGB cells were seeded at a density of 1 × 10^3^ cells per well into a 96-well plate, and then incubated in a humidified atmosphere of 95% air and 5% CO_2_ at 37°C. 24 h later, the cell culture medium was replaced before treatment with propolis or its vehicle (phenol red-free DMEM containing 1% FBS). After pretreatment with propolis or its vehicle for 1 h, a MAPK inhibitor was added to the medium separately, including SB203580 (a p38 MAPK inhibitor) and U0126 (an ERK inhibitor) at 5 *μ*M (both from Calbiochem, San Diego, CA, USA).

### 2.10. Statistical Analysis

Data are presented as means ± S.E.M. Statistical comparisons were made using Student's *t*-test or Dunnett's test or Tukey's test by means of STAT VIEW version 5.0 (SAS Institute Inc., Cary, NC, USA). A value of *P* < 0.05 was considered to indicate statistical significance.

## 3. Results

### 3.1. Effects of Propolis and Its Constituents against Visible Light-Induced Cell Damage in 661W Photoreceptor Cells

**Figure 1:**
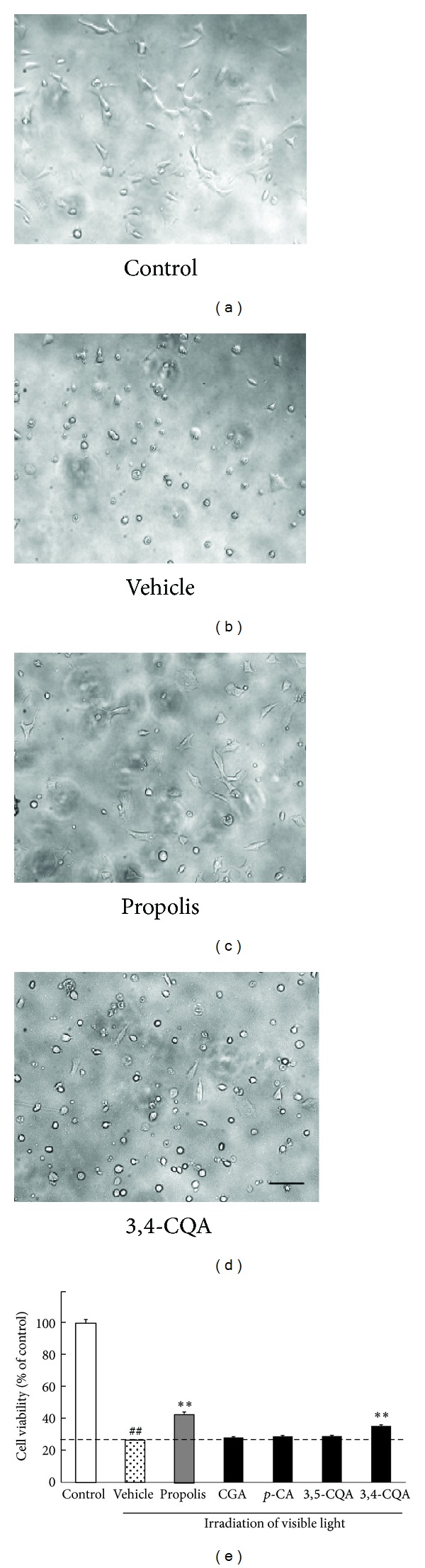
Effects of propolis and its constituent on cell damage induced by white light irradiation in a 661W culture. ((a)–(c)) Representative photographs at 24 h after light irradiation. (a) Nonirradiated cells showed a normal shape. (b) White light-induced alteration of cell shape. (c) Pretreatment with propolis and (d) pretreatment with 3,4-di-*O*-caffeoylquinic acid at 1 h before the white light irradiation recovered the cell shape, respectively. (e) Cell viability was assessed by immersing cells in WST-8 solution for 6 h at 37°C, with absorbance recorded at 492 nm. White light induced a decrease in cell viability. Propolis (30 *μ*g/mL) and 3,4-di-*O*-caffeoylquinic acid (3 *μ*g/mL) inhibited white light-induced cell damage. Data are shown as means ± S.E.M. (*n* = 6). **P < 0.01 versus light exposure plus the vehicle-treated group and ^##^ *P* < 0.01 versus control. CGA: chlorogenic acid, *p*-CA: *p*-coumaric acid, 3,5-CQA: 3,5-di-*O*-caffeoylquinic acid, 3,4-CQA: 3,4-di-*O*-caffeoylquinic acid. Scale bar represents 100 *μ*m.

We examined the effects of propolis and its constituents (chlorogenic acid, *p*-coumaric acid, 3,5-di-*O*-caffeoylquinic acid, and 3,4-di-*O*-caffeoylquinic acid) on white light-induced 661W cell damage. Representative photographs of 661W cells are shown in Figures 1(a)–1(d). As shown in Figures 1(a)–1(d), nontreated control cells displayed normal morphology (Figure 1(a)), whereas cells exposed to white light revealed shrinkage and condensation of their nuclei (Figure 1(b)). After exposure to visible light plus propolis or 3,4-di-*O*-caffeoylquinic acid, the nucleus morphology was similar to that of the normal control cells (Figures 1(a), 1(c), and 1(d)). To evaluate cell survival quantitatively, we examined the change in fluorescence intensity that occurred following the cellular reduction of WST-8 to formazan. In the white light-irradiated vehicle group, the cell viability was decreased to 30% of that of the control group. Propolis (30 *μ*g/mL) and 3,4-di-*O*-caffeoylquinic acid (3 *μ*g/mL) inhibited the decrease in cell viability by light irradiation. In contrast, chlorogenic acid, *p*-coumaric acid, or 3,5-di-*O*-caffeoylquinic acid at 3 *μ*g/mL, respectively, did not affect cell viability (Figure 1(e)).

### 3.2. Effects of Propolis and Its Constituents against UVA-Induced Cell Damage and Phosphorylated p38 MAPK in 661W Photoreceptor Cells

**Figure 2:**
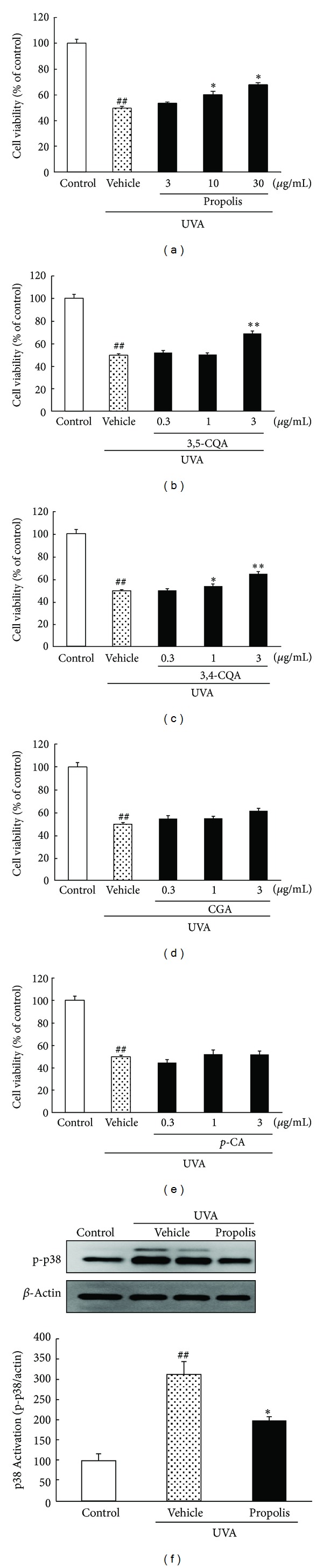
Effects of propolis and its constituents on cell damage or phosphorylated p38 induced by UVA irradiation in a 661W culture. ((a)–(e)) Cell viability was assessed by immersing cells in WST-8 solution for 6 h at 37°C, with absorbance recorded at 492 nm. UVA induced a decrease in cell viability. (a) Propolis at 10 and 30 *μ*g/mL significantly inhibited UVA-induced cell damage in a 661W culture. (b) 3,5-di-*O*-caffeoylquinic acid, (c) 3,4-di-*O*-caffeoylquinic acid, and (d) chlorogenic acid at 1 and 3 *μ*g/mL significantly inhibited cell damage, respectively. (e) p-Coumaric acid at 1 *μ*g/mL inhibited cell damage. (f) Representative band images showing activation of p38 in the nontreated, UVA exposure plus vehicle-treated, and UVA exposure plus propolis-treated cells. UVA exposure plus vehicle-treated group had 2 lanes. (g) Quantitative analysis of the band density of p38. Data are shown as means ± S.E.M. (*n* = 6). *P < 0.05, **P < 0.01 versus UVA exposure plus the vehicle-treated group, and ^##^ *P* < 0.01 versus control. CGA: chlorogenic acid, *p*-CA: *p*-coumaric acid, 3,5-CQA: 3,5-di-*O*-caffeoylquinic acid, and 3,4-CQA: 3,4-di-*O*-caffeoylquinic acid.

We studied the effects of propolis and its constituents on UVA-induced 661W cell damage. UVA irradiation at 4 J/cm^2^ induced a 0.5-fold decrease in the cell viability (versus the control group). Pretreatment with propolis at 10–30 *μ*g/mL concentration-dependently inhibited the decrease in cell viability (Figure 2(a)). The two dicaffeoylquinic acids (3,4- and 3,5-di-*O*-caffeoylquinic acid) reduced this cell damage (Figures 2(b) and 2(c)). The other chlorogenic acid and *p*-coumaric acid had no detectable effects (Figures 2(d) and 2(e)). To clarify the mechanism of action of propolis, the activities of mitogen-activated protein kinases (MAPKs), which are signals related to oxidative stress, were measured using immunoblotting. Phosphorylated p38 was markedly increased (versus nonirradiated cells) in the cells exposed to UVA, against *β*-actin. Propolis significantly reduced the UVA-induced phosphorylation of p38 (Figure 2(f)).

### 3.3. The Effect of Propolis against UVA-Induced Cell Damage in Human Skin-Derived Fibroblasts

**Figure 3:**
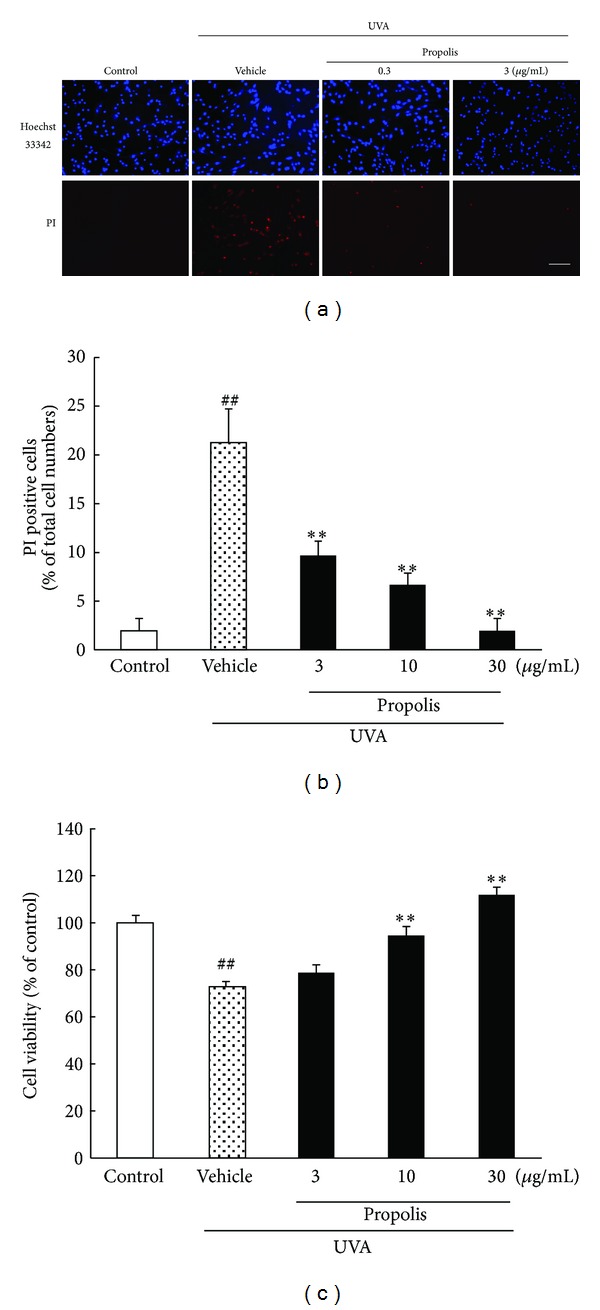
Effects of propolis on cell damage induced by UVA irradiation in an NB1-RGB culture. (a) Representative fluorescence microscopic images show nuclear staining for Hoechst 33342 and PI after UVA 10 J/cm^2^ irradiation. Upper photomicrographs show Hoechst 33342 and lower ones propidium iodide (PI) staining at 6 h after UVA irradiation. (b) The number of cells exhibiting PI fluorescence was counted, and positive cells were expressed as the percentage of PI to Hoechst 33342. Pretreatment of cells with propolis (30 *μ*g/mL) significantly reduced the amount of cell death (versus cells treated with UVA irradiation alone). (c) Cell viability was assessed by immersing cells in WST-8 solution for 6 h at 37°C, with absorbance recorded at 492 nm. UVA induced a decrease in cell viability. Propolis concentration-dependently inhibited UVA-induced cell damage. Data are shown as means ± S.E.M. (*n* = 6). *P < 0.05, **P < 0.01 versus UVA exposure plus the vehicle-treated group, and ^##^ *P* < 0.01 versus control. Scale bar represents 100 *μ*m.

Representative photographs of Hoechst 33342 and PI staining after UVA irradiation to NB1-RGB fibroblast cells are shown in Figure 3(a). Hoechst 33342 stains all cells (live and dead cells), whereas PI stains only dead cells. In the UVA 10 J/cm^2^-irradiated group, the PI positive cell numbers increased more than 10-fold (versus control). Propolis (3, 10, and 30 *μ*g/mL) added to the culture medium concentration-dependently decreased the number of cells showing PI staining after UVA irradiation (versus vehicle treatment) (Figure 3(b)). In the WST assay, cell viability was found to be reduced to 0.7-fold after UVA irradiation (versus control), and this cell damage was reduced by treatment with propolis at 3–30 *μ*g/mL in a concentration-dependent manner (Figure 3(c)).

### 3.4. Effects of Propolis Constituents against UVA-Induced Cell Damage in Human Skin-Derived Fibroblasts

**Figure 4:**
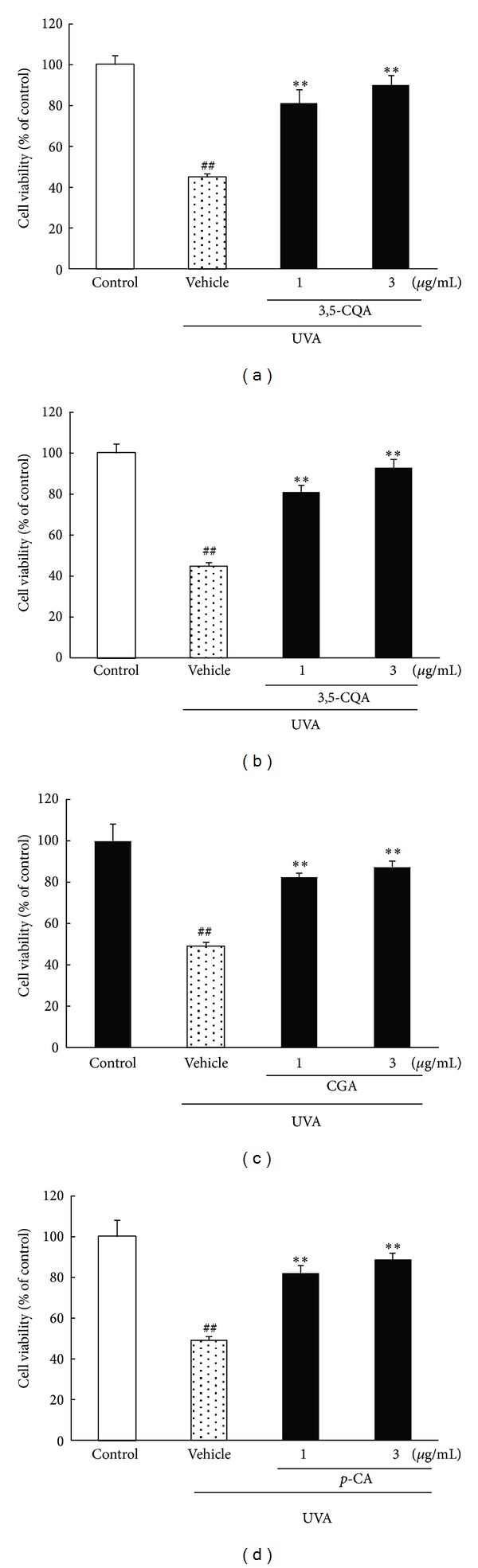
Effects of constituents of propolis on cell damage induced by UVA irradiation in an NB1-RGB culture. ((a)–(d)) Cell viability was assessed by immersing cells in WST-8 solution for 6 h at 37°C, with absorbance recorded at 492 nm. UVA induced a decrease in cell viability. (a) 3,5-Di-*O*-caffeoylquinic acid, (b) 3,4-di-*O*-caffeoylquinic acid, (c) chlorogenic acid, and (d) *p*-coumaric acid at 1 and 3 *μ*g/mL significantly inhibited cell damage, respectively (versus cells treated with UVA irradiation alone). Data are shown as means ± S.E.M. (*n* = 6). **P < 0.01 versus UVA exposure plus the vehicle-treated group and ^##^ *P* < 0.01 versus control. CGA: chlorogenic acid, *p*-CA: *p*-coumaric acid, 3,5-CQA: 3,5-di-*O*-caffeoylquinic acid, and 3,4-CQA: 3,4-di-*O*-caffeoylquinic acid.

As described above, propolis has protective effects against UVA-induced cell damage in NB1-RGB cells. We next studied the effects of four constituents of propolis. In the UVA 10 J/cm^2^-irradiated group, cell viability decreased 0.5-fold (versus control) (Figure 4). All four constituents suppressed this decrease in cell viability in a concentration-dependent manner, its effect being significant at concentrations of 3 *μ*g/mL or more (Figures 4(a)–4(d)).

### 3.5. Effect of Propolis on UVA- or White Light-Induced Intracellular ROS Production in Human Skin-Derived Fibroblasts or 661W Photoreceptor Cells

**Figure 5:**
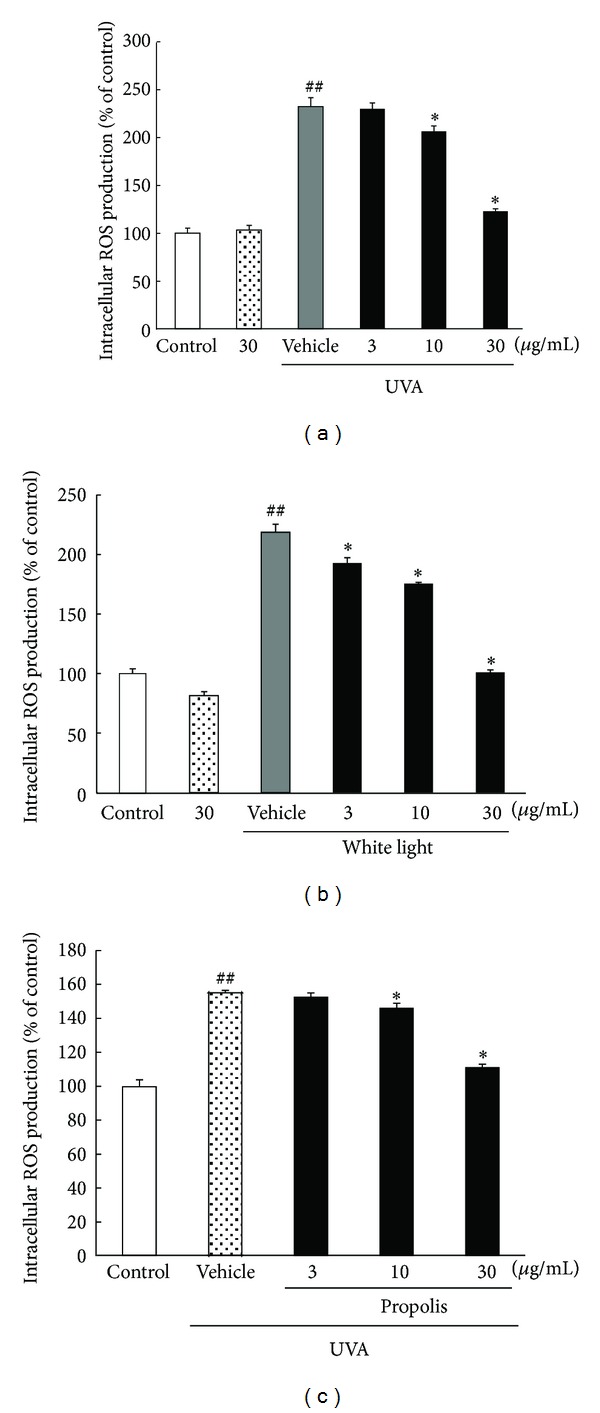
Effects of propolis on intracellular ROS production induced by UVA or white light irradiation in an NB1-RGB or 661W culture. The NB1-RGB or 661W culture was treated with propolis for 1 h, and this was supplemented with CM-H_2_DCFDA at 10 *μ*M for 20 min. ROS production was stimulated with UVA 10 J/cm^2^ or 3,000lx of white light, and fluorescence was measured at 0–20 min. UVA or white light irradiation induced oxidation of DCFH in NB1-RGB or 661W culture. Intracellular ROS production was increased by UVA or white light irradiation, while it was concentration-dependently reduced by propolis treatment. Data are shown as means ± S.E.M. (*n* = 6). *P < 0.05, **P < 0.01 versus UVA or white light exposure plus the vehicle-treated group, and ^##^ *P* < 0.01 versus control.

To investigate the inhibitory effect of propolis on intracellular ROS production by UVA or white light irradiation in NB1-RGB or 661W cells, we employed a radical scavenging-capacity assay using the ROS-sensitive probes 5-(and-6-)chloromethyl-2′,7′-dichlorodihydrofluorescein diacetate (CM-H_2_DCFDA). In the 661W photoreceptor cells UVA 10 J/cm^2^ or 3,000lx of white light-irradiated group, the intracellular ROS production increased 2.5- or 2.3-fold, respectively (versus control), and it was concentration-dependently suppressed by the addition of propolis (Figures 5(a) and 5(b)). In the human skin-derived fibroblasts UVA 10 J/cm^2^-irradiated group, the intracellular ROS production increased 1.5-fold (versus control), and it was concentration-dependently suppressed by the addition of propolis (Figure 5(c)).

### 3.6. Phosphorylations of p38 and ERK Induced by UVA and the Effects of an MAPK Inhibitor in Human Skin-Derived Fibroblasts

**Figure 6:**
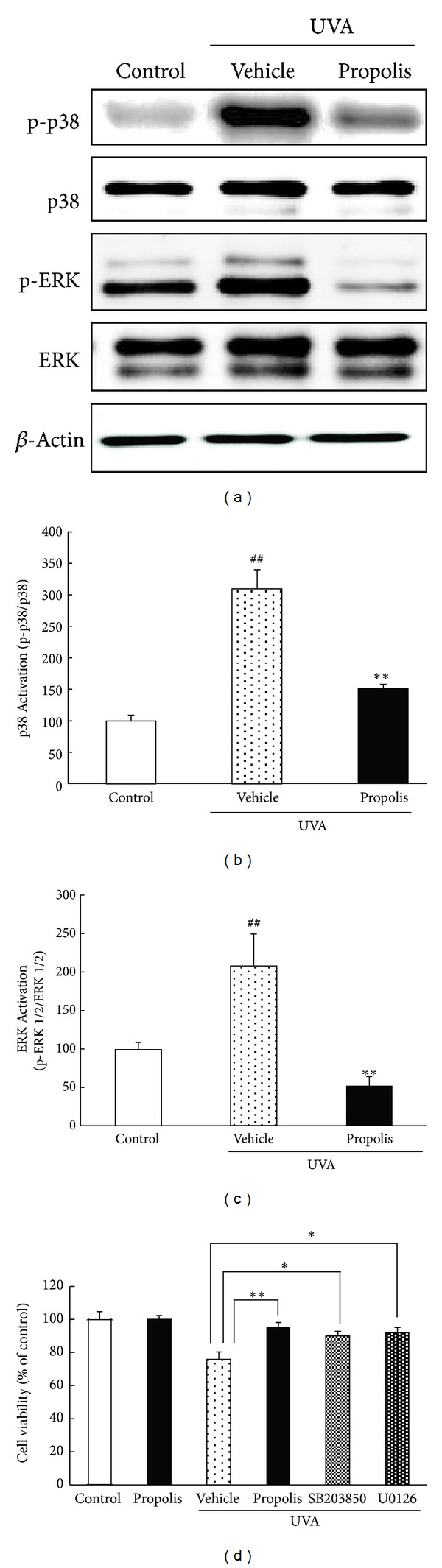
Effects of propolis on UVA-induced expression of phosphorylated p38 and ERK1/2 and MAPK inhibitors in an NB1-RGB culture. Representative Western blots (a) showing activation of p38 and ERK in the nontreated, UVA 10 J/cm^2^ exposure plus vehicle-treated, and UVA exposure plus propolis-treated (30 *μ*g/mL) groups. Phosphorylation of p38 (b) and ERK1/2 (c) was determined by immunoblotting assay. Quantitative analysis of the band density of p38 (b) and ERK (c) by densitometric analysis. These intensities were normalized with total p38 and total ERK, respectively. (d) Cell viability was assessed by immersing cells in WST-8 solution for 6 h at 37°C, with absorbance recorded at 492 nm. SB203580 and U0126 inhibited the decrease of cellular viability induced by UVA, and the level was as high as of a propolis-treated group. Data are shown as means ± S.E.M. (*n* = 6). **P < 0.01 versus UVA exposure plus vehicle-treated cells and ^##^ *P* < 0.01 versus control.

To investigate the mechanism by which propolis suppressed cell damage by UVA, we evaluated the activities of mitogen-activated protein kinase (MAPKs), which are signals related to oxidative stress, stimulated by UVA irradiation in NB1-RGB cells using Western blotting. Phosphorylated-p38 (p-p38) and phosphorylated-extracellular signal regulated protein kinases (p-ERK1/2) were markedly increased (versus control) in NB1-RGB cells that were irradiated 10 J/cm^2^ UVA, against total p38 and ERK, respectively. UVA irradiation increased the levels of p-p38 and p-ERK by 2.0- and 3.0-fold, respectively. Propolis (30 *μ*g/mL) treatment significantly reduced the UVA-induced phosphorylation of p38 and ERK (Figures 6(a)–6(c)). Treatment with SB203580 (a p38 MAPK inhibitor) or U0126 (an ERK inhibitor) at 1 h before UVA irradiation inhibited the decrease of cellular viability induced by UVA (Figure 6(d)). Although we have examined whether an MAPK inhibitor could alter the effects of propolis, an MAPK inhibitor did not affect the cell viability of a propolis-treated group.

## 4. Discussion

In the present study, we demonstrated that propolis and its constituents suppressed cell damage induced by white light or UVA in 661W cells and by UVA in NB1-RGB cells. Treatment with propolis suppressed the intracellular ROS production stimulated by UVA or white light irradiation. Propolis also inhibited the UVA-induced phosphorylation of p38 and ERK.

Brazilian green propolis (water-extract propolis; WEP) exhibited potent antioxidant effects against a variety of ROS in our previous report [29]. Moreover, it has been reported that the main constituents of WEP (caffeoylquinic acid derivatives: 3,4-CQA, 3,5-CQA) were also found to have antioxidant effects with similar efficacies to those of trolox, which is a major antioxidant [29]. These constituents may be mainly responsible for the powerful antioxidative effects of WEP. In the present study, propolis and its constituents (3,4-CQA and 3,5-CQA) suppressed cell damage induced by UVA irradiation via antioxidant effects. Surprisingly, CGA and *p*-CA also inhibited UVA-induced cell damage. It is suggested that *p*-CA with IC_50_ values of more than 100 *μ*M did not scavenge any of the ROS [29]. It has been suggested that antioxidant properties arise from complex mechanisms or synergistic interactions between constituents of propolis. However, further studies are necessary to understand the exact protective mechanism of CGA and *p*-CA.

UV irradiation gives rise to the activation of multiple cell surface cytokine and growth factor receptors, mitogen-activated protein kinases (MAPKs), signal modules such as extracellular-regulated protein kinase (ERK), and p38 kinase [30]. Fibroblasts exposed to UV-induced oxidative stress have been shown to increase the expression of phosphorylated-MAPKs (p38 and ERK) [30, 31]. Various stresses, including ischemia, ultraviolet exposure, and oxidative stress stimulate p38 and JNK activation [32]. They are involved in cell apoptosis and differentiation [32]. Rapid activation of p38 can be induced by a variety of cellular stressors, including UV irradiation. We investigated whether propolis could inhibit UV-induced phosphorylation of MAPKs. The present data were consistent with studies by Syed et al. [33], where it was shown that the peak of the activated p38 in normal keratinocytes is 2–10 min after UVA irradiation. These results indicate that UVA-induced ROS production may cause the activation of a p38 signaling cascade, and propolis can reduce UVA-induced fibroblasts damage by an antioxidative mechanism. Moreover, oxidative stress, mitogens, and survival factors activate ERK activation. ERK is involved in cell proliferation and differentiation [34]. ERK activation achieved a peak immediately after UVA irradiation, and propolis significantly inhibited ERK activation. Taken together, the activation of ERK may provide a survival signal that allows human fibroblasts to escape from UVA-induced apoptosis. Although MAPKs (p38 and ERK) are activated by UVA irradiation, the effects of their inhibitors on UVA-induced human skin-derived fibroblasts cells damage are still unknown. SB203580 (a p38 inhibitor) and U0126 (an ERK inhibitor) inhibited the decrease of cellular viability induced by UVA, and the level was as high as of a propolis-treated group. Our data indicate that p38 and ERK signal pathways are involved in UVA-induced cellular damage and there is a potential link between propolis and MAPK, suggesting that MAPK signaling is involved in UVA damage and propolis shows the protective effects by suppressing the MAPK activation.

Activator protein-1 (AP-1) is an important factor for UVA-induced skin damage. AP-1 is composed of heterodimers of members of the Fos (c-Fos, FosB, Fra-1, and Fra-2) and the Jun (c-Jun, JunB, and JunD) families of proteins or of homodimers of members of the Jun family of proteins [35]. AP-1 induces collagen degradation by promoting the expression of matrix metalloproteinases MMP-1, MMP-3, and MMP-9 [36, 37]. Furthermore, AP-1 causes collagen degradation by preventing the expression of procollagen-1 [38]. Reports suggest that AP-1 activation through p38 and/or ERK and/or JNK (c-Jun N-terminal kinase) is essential for UVA-induced skin damage. Therefore, there is a possibility that propolis may inhibit AP-1 activation by suppressing p38, ERK, and JNK activation. However, further studies will be needed to clarify the precise mechanisms in UVA-induced skin damage.

## 5. Conclusion

Propolis may become a therapeutic candidate for the treatment of AMD and skin damage induced by visible light or UV irradiation.
